# Pancreatic α-cells integrate immune and neuronal stimuli for insulin secretion

**DOI:** 10.1016/j.celrep.2026.117586

**Published:** 2026-06-22

**Authors:** Kelly A. Trimigliozzi, Sophia J. Wiedemann, Giada Ostinelli, Leila Rachid, Hélène Méreau, Marianne Böni-Schnetzler, Guy A. Rutter, Daniel T. Meier, Marc Y. Donath

**Affiliations:** 1 Department of Biomedicine, University of Basel, Basel, Switzerland; 2 Clinical Trial Unit, Cantonal Hospital Baden, Baden, Switzerland; 3 Research Centre (CRCHUM) and University Hospital, University of Montreal, Montreal, QC, Canada; 4 Department of Metabolism, Digestion and Reproduction, Faculty of Medicine, Imperial College London, London, UK; 5 Lee Kong Chian School of Medicine, Nanyang Technological University, Singapore, Singapore; 6 Research Institute of the McGill University Hospital Center, Montréal, QC, Canada

## Abstract

Insulin secretion can be stimulated by immune and neuronal processes prior to a rise in blood glucose, exemplified by the cephalic phase of insulin response in the anticipation of food. Pancreatic α-cells prevent hypoglycemia by releasing glucagon. Here, we identified α-cells as critical mediators of IL-1β- and cholinergic agonist-driven insulin secretion. Cholinergic blockade prevented glucagon-stimulated insulin secretion in mice. Selective ablation of α-cells abolished cephalic phase insulin release. Islets from α-cell-deficient mice also failed to secrete insulin in response to IL-1β or muscarinic receptor activation. However, glucagon, acting on glucagon and GLP-1 receptors, rescued this insulin-stimulatory response. Mechanistically, intracellular Ca^2+^ mobilization at fasting glucose mediated cholinergic and IL-1β-stimulated insulin release. Short-term high-fat diet impaired glucagon-induced insulin secretion *in vivo,* while isolated islets showed increased insulin secretion after cholinergic stimulation versus chow-fed controls. These findings reveal α-cell-derived glucagon as a gatekeeper of immune and neuronal control of insulin secretion at fasting glucose.

## In brief

Trimigliozzi et al. show that α-cells integrate neuronal and immune signals to release glucagon, which acts on β-cell glucagon and GLP-1 receptors to drive insulin secretion at fasting glucose concentrations. Their findings reveal that functional α-cells are required for responding to neuronal or immune stimulation under low-glucose conditions.

## Graphical abstract

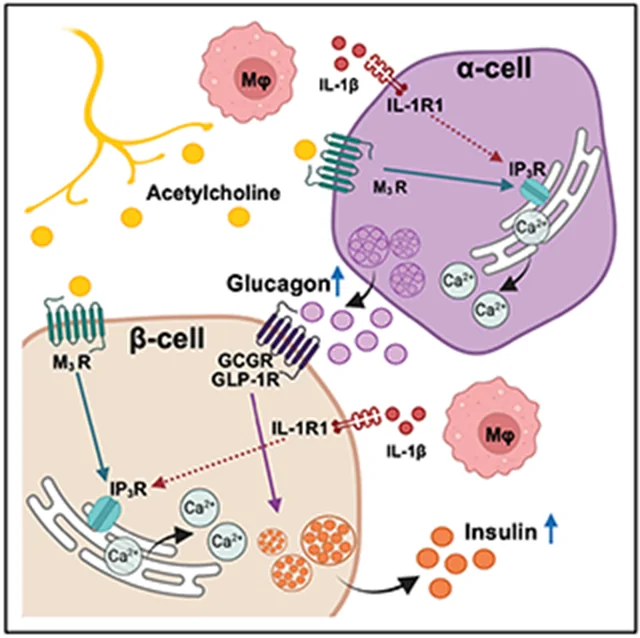

## INTRODUCTION

Pancreatic islets are richly innervated by the parasympathetic nervous system, and their neurotransmitter acetylcholine enhances glucose-stimulated insulin secretion via muscarinic receptors.^1–5^ However, certain neuronally driven insulin secretory processes, such as the cephalic phase insulin response (CPIR), occur independently of a rise in blood glucose.^6^

CPIR is a rapid, anticipatory release of insulin, triggered by sensory cues at meal onset and precedes changes in blood glucose levels.^6,7^ CPIR is essential for priming metabolic tissues to maintain postprandial glucose homeostasis.^8–10^ Disruption of this pathway, such as bypassing the oral cavity, leads to impaired glucose tolerance.^11–13^ Conflicting results exist regarding the CPIR during metabolic stress, wherein some report attenuated responses,^14,15^ while others describe unchanged or even heightened responses in obese individuals.^16–18^

Chronically increased activation of the inflammasome-IL-1β pathway is observed in obesity and contributes to the development of type 2 diabetes and its complications.^19,20^ In contrast, the acute physiological postprandial release of IL-1β from myeloid cells enhances insulin secretion, akin to the incretin effect.^21^ Interestingly, IL-1β has recently emerged as a mediator of the CPIR.^15^ Notably, diet-induced obesity impairs CPIR in mice, the underlying cause being dysfunctional IL-1β signaling.^15^

Pancreatic α-cells also regulate insulin secretion.^22–25^ Beyond preventing hypoglycemia by stimulating hepatic glucose production,^26^ glucagon derived from α-cells has also been reported to rapidly stimulate insulin secretion prior to changes in systemic blood glucose levels.^27^ Basal glucagon secretion is also regulated by the parasympathetic nervous system.^28–30^ Indeed, systemic glucagon levels increase following cephalic stimulation in dogs,^31^ and oral sensory stimuli elevate glucagon in healthy humans.^32^ Furthermore, human α-cells may contribute to insulin secretion not only via glucagon but also by releasing acetylcholine.^33^

We hypothesized that pancreatic α-cells mediate neuronally and immunologically driven insulin secretion in the absence of stimulation by glucose. In this study, we found that glucagon-stimulated insulin secretion was attenuated upon muscarinic receptor inhibition. We identified α-cell-derived glucagon as a key regulator of both neuronal and IL-1β-induced insulin secretion. Furthermore, we demonstrated that short-term high-fat diet (HFD) feeding disrupts this α-cell-β-cell interaction and heightens β-cell responsiveness to cholinergic stimulation. These findings position α-cells as integrators of neuronal- and immune-stimulated insulin secretion.

## RESULTS

### α-cells are essential for neuronal and IL-1β-stimulated insulin secretion

The role of glucagon as an insulin secretagogue was first demonstrated in healthy, lean individuals.^27^ To recapitulate this phenomenon in animals, we injected glucagon in overnight-fasted wildtype mice, which triggered an acute rise in circulating insulin levels prior to detectable changes in blood glucose (Figures 1A and 1B; Figure S1A). This insulinotropic effect was abolished by pretreatment with atropine (Figures 1C and 1D; Figure S1B), suggesting that glucagon-stimulated insulin secretion relies on parasympathetic activity. Pretreatment with the GLP-1 receptor (GLP-1R) antagonist exendin-9-39 (Ex-9-39) also attenuated glucagon-stimulated insulin secretion (Figures 1E and 1F; Figure S1C), suggesting that glucagon acts directly on pancreatic β-cells to promote insulin secretion at fasting glucose levels, at least in part via GLP-1R signaling. Acute IL-1β treatment induces insulin secretion upon glucose stimulation.^15,21,34^ We found that IL-1β also stimulates insulin release in overnight-fasted mice (Figure 1G). Moreover, we observed increased systemic glucagon levels after IL-1β treatment and no changes in blood glucose levels (Figures 1H and 1I; Figure S1D). To delineate the contribution of the parasympathetic nervous system to IL-1β-induced endocrine hormone release, we pretreated mice with atropine prior to IL-1β administration. Atropine treatment did not abolish IL-1β-stimulated insulin secretion (Figure S1E), though the magnitude of the response was attenuated relative to atropine-naïve mice (Figure 1G, 87% increase above saline in atropine-naïve; Figure S1E, 50% increase above saline in atropine-pretreated mice). By contrast, IL-1β-stimulated glucagon secretion was suppressed by atropine pretreatment (Figure S1F). Although blood glucose levels did not differ between treatment groups (Figures S1G and S1H), we noted that absolute glycemia was modestly higher in atropine-pretreated mice (Figure S1G, IL-1β-treated = 5.6mM) compared to atropine-naïve mice (Figure 1I, IL-1β-treated = 3.8mM). Given that α-cell glucagon release is known to be strongly suppressed at glucose concentrations of 5-7mM,^35,36^ the blunted glucagon response we observed with atropine is at least partly attributable to this modestly elevated glycemia, which in turn likely contributed to the attenuated IL-1β-stimulated insulin secretion. Taken together, these results suggest that cholinergic signaling and α-cell-derived glucagon contribute to the full magnitude of IL-1β-induced insulin release.

To test this hypothesis, we employed an inducible transgenic mouse model with diphtheria toxin-induced ablation of α-cells (α-KO^37^). IL-1β-induced insulin secretion was markedly reduced in α-KO mice compared to their wildtype littermates, with no changes in blood glucose levels (Figures 1J and 1K; Figure S1I), implicating α-cells as critical mediators of IL-1β-stimulated insulin release in overnight-fasted mice. Given prior evidence that IL-1β mediates cephalic phase insulin release,^15^ we next assessed whether α-cells contribute to cephalic phase insulin secretion as well. While circulating glucagon levels were unchanged during cephalic stimulation (Figures S1J–S1L), α-KO mice exhibited a near-complete loss of the CPIR (Figures 1L and 1M; Figure S1M). Taken together, these findings show that α-cells are indispensable for immune and neuronally mediated insulin secretion under fasting glucose conditions.

### α-cell secreted glucagon is essential for IL-1β- and cholinergic-induced insulin secretion

To delineate the role of α-cells in insulin secretion further, we isolated islets and cultured them in low-glucose media (3.5 mM), at the threshold of hypoglycemia,^38^ to mimic fasting conditions. Stimulating islets with carbachol, a muscarinic receptor agonist, elicited glucagon release (Figure 2A; Figures S2A and S2B) and an increase in insulin secretion (Figures 2B, S2C and S2D). In contrast to our *in vivo* observations (Figures 1G and 1H), IL-1β alone failed to induce either glucagon or insulin secretion *in vitro* (Figures 2A and 2B). Since isolated islets lack neuronal input, we next co-stimulated the islets with IL-1β and carbachol, which led to increased release of both hormones (Figures 2A and 2B).

Further, compared to islets from their wildtype littermates, α-KO islets exhibited a strongly suppressed IL-1β and carbachol-stimulated insulin secretion (Figures 2C, S2E and S2F), suggesting that α-cells are essential for this stimulation. Supplementing glucagon into the media of α-KO islets restored insulin secretion under IL-1β and carbachol stimulation (Figures 2D, S2G and S2H). By contrast, supplementing exendin-4, a GLP-1 agonist, failed to restore IL-1β and carbachol-stimulated insulin secretion (Figures 2E, S2I and S2J), indicating that the underlying signaling cascade is glucagon-dependent. Pharmacologically blocking the glucagon receptor with the antagonist Adomeglivant (GRA) and the GLP-1R antagonist Ex-9-39 strongly hampered carbachol and IL-1β-induced insulin release, yet there was no synergistic effect from blocking both receptors (Figures 2F, S2K and S2L). These results point to α-cell-derived glucagon as a critical mediator of cholinergic and IL-1β-induced insulin secretion.

### Intracellular Ca^2+^ stores are mobilized by immune and neuronally stimulated insulin secretion

We next investigated the intracellular signaling mechanisms underlying carbachol- and IL-1β-induced insulin secretion at fasting glucose conditions (3.5 mM). Islets from mice expressing the genetic Ca^2+^ sensor GCaMP6f specifically in the cytoplasm of β-cells (*Ins1Cre-GcAMP6*^*fl/fl* 39^) *w*ere imaged for 20 min, during which treatments were applied after an initial 5-min baseline period. Whole islet recordings revealed that IL-1β alone did not affect intracellular Ca^2+^ dynamics (Figure 3A). Carbachol, which stimulates the release of Ca^2+^ from intracellular calcium stores via the inositol triphosphate receptor (IP3R),^4^ increased cytoplasmic Ca^2+^ concentrations, and co-stimulation with IL-1β further elevated peak Ca^2+^ levels (Figure 3A). All islets treated with carbachol or IL-1β combined with carbachol were responsive (Figure S3A), displaying Ca^2+^ levels that were at least 20% above baseline. Moreover, both treatments elicited Ca^2+^ responses within the first 5 min after stimulation (Figure 3B). The first peak amplitude tended to be higher in IL-1β and carbachol-treated islets (0.892 ± 0.572) compared with carbachol alone (0.611 ± 0.305, *p* = 0.09) (Figure 3C), yet the total Ca^2+^ signal over the entire recording period was similar between the two groups (Figure S3B). These findings indicate that intracellular Ca^2+^ levels are elevated in islets treated with carbachol under fasting glucose conditions and that IL-1β provides a modest additional enhancement of this response.

To assess the contribution of extracellular Ca^2+^ influx, we treated wildtype islets with diazoxide, a KATP channel opener that prevents membrane depolarization and subsequent activation of voltage-gated calcium channels.^40^ We first confirmed an inhibitory dose of diazoxide for glucose-stimulated insulin secretion (Figures S3C–S3E). At 3.5mM glucose condition, diazoxide suppressed IL-1β and carbachol-induced glucagon and insulin secretion, yet insulin levels remained significantly elevated compared to unstimulated islets (Figures 3D and 3E; Figures S3F–S3I). Next, we directly blocked Ca^2+^ influx by treating islets with verapamil, a calcium channel inhibitor.^41^ While verapamil markedly inhibited glucose-stimulated insulin secretion (Figures S3J–S3L), it enhanced IL-1β and carbachol-stimulated insulin release (Figures 3G, S3O and S3P). Similarly, glucagon secretion was modestly increased in the presence of IL-1β, carbachol, and verapamil (Figures 3F, S3M and S3N). Taken together, these data demonstrate that Ca^2+^ influx plays a minor role in neuronal and IL-1β-stimulated glucagon and insulin secretion at fasting glucose levels.

Based on these findings, we reasoned that although an increase in intracellular Ca^2+^ is necessary for carbachol and IL-1β-stimulated insulin secretion, the underlying mechanism likely involves the mobilization of intracellular Ca^2+^ stores. To test this hypothesis, we inhibited the IP3R, which is highly expressed on the endoplasmic reticulum (ER) of β-cells,^42^ by administering 2-aminoethoxydiphenyl borate (2-APB). Blocking intracellular Ca^2+^ release from the ER prevented the cholinergic potentiation of insulin secretion at high glucose (Figures S4A–S4C). At fasting glucose levels, islets treated with 2-APB had strongly reduced carbachol and IL-1β-induced glucagon and insulin secretion (Figures 3H and 3I; Figures S4D–S4G). To confirm that extracellular Ca^2+^ influx does not contribute to the stimulatory action of carbachol and IL-1β, experiments were repeated in Ca^2+^-free media (Figures S4H–S4M). Under these conditions, 2-APB still robustly suppressed glucagon and insulin secretion, demonstrating that its inhibitory effect is not amplified by a lack of extracellular Ca^2+^, thereby indicating that IP3R-mediated Ca^2+^ release is essential for this response. To determine whether the suppression of IL-1β and carbachol-stimulated insulin secretion was due to impaired mobilization of intracellular Ca^2+^ stores rather than reduced glucagon release, we supplemented glucagon into the media. We found that 2-APB robustly inhibited immune and neuronally driven insulin secretion at fasting glucose levels, even when glucagon was supplemented (Figures 3J, S4N and S4O). Furthermore, Ex-4 supplementation also failed to restore IL-1β- and carbachol-stimulated insulin secretion in the presence of 2-APB (Figures 3K, S4P and S4Q). Considering that both glucagon and Ex-4 are known to potentiate glucose-stimulated insulin secretion, we next tested whether IP3R blockade would similarly impair immune- and cholinergic-stimulated insulin secretion at elevated glucose concentrations (16.7mM). Despite high glucose, 2-APB robustly attenuated the potentiation of insulin secretion by IL-1β and carbachol, and this inhibition could not be rescued by the addition of exogenous glucagon or exendin-4 (Figures S4R–S4T). These findings indicate that neuronal and IL-1β-stimulated insulin secretion depends on the mobilization of intracellular Ca^2+^ stores.

### Short-term HFD feeding disrupts α-cell-stimulated insulin secretion *in vivo*, yet increases cholinergic potentiation of insulin secretion *ex vivo*

In contrast to chow-fed mice (Figure 1A), an acute intravenous glucagon injection failed to induce insulin secretion in mice fed HFD for 5 weeks (Figures 4A and 4B; Figure S5A). On the other hand, acute IL-1β administration still elicited both insulin and glucagon release while on HFD (Figures 4C–4E; Figure S5B). This indicates that the stimulatory effects of IL-1β on endocrine hormone release are sustained even during HFD feeding, yet exogenous glucagon no longer elicits insulin secretion in the fasted state.

To determine whether HFD alters the capacity of islets to respond to IL-1β and carbachol at fasting glucose levels, we isolated islets from HFD-fed mice and stimulated them *ex vivo*. Consistent with the findings in lean mice (Figures 2A and 2B), IL-1β alone failed to stimulate either glucagon or insulin release at 3.5mM glucose (Figures 4F and 4G; Figures S5C–S5F). Carbachol significantly increased glucagon secretion, which was further potentiated by co-stimulation with IL-1β. Strikingly, carbachol alone induced a 7-fold increase in insulin secretion in islets from HFD-fed mice (Figure 4G), a response markedly greater than the 2-fold increase observed in chow-fed mice (Figure 2B). Moreover, combined IL-1β and carbachol treatment in islets of HFD-fed mice elicited an even more pronounced 14-fold increase in insulin secretion (Figure 4G). These findings indicate that HFD feeding renders *ex vivo* pancreatic β-cells highly responsive to muscarinic stimulation, even at fasting glucose levels.

## DISCUSSION

While the parasympathetic nervous system has long been recognized as the primary driver of CPIR,^5^ the intermediate cellular mechanisms remain elusive. We show that α-cell-derived glucagon is required for insulin secretion in response to both muscarinic stimulation and IL-1β at fasting glucose levels. Glucagon-stimulated insulin secretion was abolished by muscarinic blockade, and mice lacking α-cells did not mount the CPIR. Modeling cephalic stimulation *in vitro* further revealed that muscarinic and IL-1β signaling increase both glucagon and insulin secretion, but these responses were lost in α-cell-deficient islets. Only glucagon, and not GLP-1, restored insulin secretion under these conditions, suggesting that glucagon is the principal α-cell-derived mediator of cholinergic and immune-induced β-cell activation. Mobilization of intracellular calcium stores is needed for cholinergic and IL-1β-stimulated insulin secretion. Short-term HFD feeding disrupted glucagon-stimulated insulin secretion *in vivo*. In contrast, metabolic stress rendered β-cells highly responsive to muscarinic stimulation *ex vivo*, while the magnitude of glucagon secretion was unchanged, pointing toward a discrepancy in β-cell sensitivity to cholinergic inputs under metabolic stress.

We demonstrated that intravenous glucagon administration increased circulating insulin levels in mice before any detectable changes in blood glucose. This aligns with classic observations of glucagon as an insulin secretagogue in humans.^27^ Atropine-mediated blockade of cholinergic signaling abolishes glucagon-induced insulin secretion, linking parasympathetic inputs with glucagon-stimulated insulin release. Similarly, patients who have undergone truncal vagotomy exhibit impaired glucagon-stimulated C-peptide responses.^43^ Consistent with a role of cholinergic signaling promoting glucagon release, selective activation of G_q_ signaling in α-cells, which is the same G protein downstream of muscarinic receptor M3, stimulates glucagon secretion and increases insulin release *in vivo.*^44^ Further supporting a role of α-cells in neuronally driven insulin secretion, we found that α-KO mice have a blunted CPIR. Positioning α-cells as gatekeepers of the CPIR provides a fail-safe mechanism since the release of insulin during fasting could cause hypoglycemia, yet the simultaneous secretion of glucagon ensures that a counter-regulatory hormone is present, thereby safeguarding against insulin-induced hypoglycemia.

Previous studies suggested that glucagon alone is unable to stimulate insulin secretion at low glucose,^24^ yet this conclusion was drawn from perfused pancreas models, where anesthetized animals may lack parasympathetic input.^45^ Glucagon and GLP-1, two key α-cell-derived peptides, are known to modulate β-cell function.^25,46–48^ Specifically, glucagon acting via both GCGR and GLP-1R, enhances cAMP signaling in β-cells, thereby tuning their responsiveness to stimuli such as glucose.^22,49^ We found that specifically, glucagon mediates cholinergic and IL-1β-induced insulin secretion at fasting glucose levels via both GCGR and GLP-1R.

The differing downstream signaling of the GCGR and the GLP-1R may explain why glucagon uniquely enables insulin secretion at fasting glucose levels. Zhang et al.^36^ demonstrated that GCGR activation mobilizes adenylate cyclase (AC) 5 in β-cells, the same AC isoform engaged by high glucose, whereas GLP-1R signaling recruits a different AC pool. The engagement of AC5 downstream of the GCGR may therefore be necessary for the carbachol and IL-1β-stimulated insulin secretion at fasting glucose, a signal that GLP-1R agonism alone cannot provide. Furthermore, glucagon action on the GLP-1R may itself be mechanistically distinct from that of GLP-1. Tong et al.^49^ recently showed that β-cells adjacent to α-cells have GLP-1R-enriched nanodomains at the β-cell membrane, rendering β-cells highly sensitive to micromolar concentrations of glucagon. Importantly, α-cell activity drives the pre-internalization of these GLP-1R nanodomains at low glucose, which in turn primes β-cell Ca^2+^ response to subsequent stimuli such as glucose. Taken together, these findings suggest that the unique capacity of glucagon to engage AC5 activation downstream of the GCGR and activate GLP-1R enriched nanodomains may contribute to its insulinotropic effect and positions specifically glucagon as the main α-cell-derived mediator of immune- and cholinergic-stimulated insulin secretion at fasting glucose conditions.

We found that acute *in vivo* IL-1β stimulation robustly increased both glucagon and insulin secretion. Pancreatic islets express high levels of IL-1 receptor type 1 (IL-1R1), compared to other tissues.^50^ Within the islets, β-cells have the highest expression of IL-1R1, while non-insulin-expressing endocrine cells, including α-cells, express the receptor at lower levels.^51^ Islet resident macrophages are the most likely source of IL-1β. Interestingly, they display higher *Il1b* transcript levels than other tissue resident macrophages.^52^ However, in *ex vivo* experiments, IL-1β stimulation alone did not induce insulin or glucagon secretion from isolated islets, nor did it alter intracellular Ca^2+^ dynamics in β-cells. This discrepancy may be explained by the absence of neural inputs in *ex vivo* cultured islets,^53^ suggesting that the insulinotropic effect of IL-1β requires additional stimuli, such as cholinergic stimulation. This is in line with our previous observation that IL-1β enhances vagal nerve activity at basal glucose levels *in vivo*,^15^ suggesting that neural elements upstream of the islet represent an additional site of IL-1β action. Considering the high IL-1R1 expression in islets, we propose that IL-1β acts both at the level of the vagus nerve and endocrine cells of the islet. Although β-cells may respond directly to IL-1β, the dependence of α-cell-derived glucagon for IL-1β-stimulated insulin secretion in our model suggests that β-cell IL-1R1 signaling is not sufficient in isolation to induce insulin secretion at fasting glucose conditions.

In line with this, cytokines such as IL-1β have been shown to modulate Ca^2+^ handling in β-cells.^54^ Although IL-1β alone had no detectable effect on intracellular Ca^2+^ under our experimental conditions, co-stimulation with IL-1β and carbachol tended to elicit a higher Ca^2+^ peak than carbachol alone, indicating a subtle enhancement in Ca^2+^ dynamics. Since our Ca^2+^ recordings were restricted to β-cells (*Ins1Cre-GcAMP6*), we cannot exclude the possibility that IL-1β may also modulate Ca^2+^ dynamics in α-cells, thereby influencing glucagon release and ultimately insulin secretion. While future studies will be needed to explore this mechanism, IL-1R1 activation may enhance muscarinic receptor subtype 3 (M3R) signaling through downstream protein kinase pathways,^55^ potentially by enhancing phospholipase activation or increasing IP3R sensitivity to IP3. Importantly, these acute effects likely differ from the chronic action of IL-1β, which over hours to days can impair β-cell function and reduce viability.^56,57^

Indeed, the observed acute physiological stimulation of insulin secretion by IL-1β at fasting glucose levels may appear to contradict the well-established pathological role of chronically elevated IL-1β in the development and progression of type 2 diabetes.^19,20^ However, elevated fasting insulin levels are commonly observed in individuals with prediabetes and have been proposed to contribute to obesity and subsequent progression to diabetes.^58^ This aligns with our previous observation that HFD-induced elevation of IL-1β enhances basal insulin secretion while compromising neuronally driven insulin secretion.^15^

Mechanistically, our data indicate that the mobilization of Ca^2+^ from ER stores is essential for IL-1β- and cholinergic-stimulated glucagon and insulin release under fasting glucose conditions, as blocking IP3R with 2-APB abolished this response. Of note, chronic cytokine treatment has been shown to increase ER Ca^2+^ storage,^56^ but this adaptation is unlikely to explain the rapid responses observed in our experiments. Furthermore, cholinergic stimulation alone triggered glucagon secretion, which was further potentiated by co-stimulation with IL-1β, aligning with recent evidence that ER calcium fluxes regulate glucagon secretion at fasting glucose.^59^ Nonetheless, given the limited selectivity of 2-APB,^60^ we cannot exclude potential contributions from other Ca^2+^ entry pathways or ion channels.^39^

HFD-fed mice exhibited impaired glucagon-stimulated insulin secretion, while IL-1β-stimulated glucagon and insulin secretion remained intact. This suggests that, under conditions of diet-induced obesity, IL-1β-mediated insulin secretion no longer depends on additional glucagon signaling, possibly by the direct action of IL-1β on the vagus nerve,^15^ a mechanism that may persist despite HFD-induced metabolic dysfunction. Indeed, the hyperinsulinemia observed in obesity has been suggested to be driven by heightened parasympathetic tone.^61–63^ Additionally, β-cells of long-term HFD-fed mice exhibit increased sensitivity to acetylcholine, which may contribute to hyperinsulinemia.^64–66^ In line with this, we found that islets isolated from HFD-fed mice displayed markedly increased insulin secretion in response to carbachol stimulation at fasting glucose levels, an effect which was further potentiated by IL-1β co-stimulation. In contrast, glucagon secretion in response to carbachol and IL-1β was not augmented. On the other hand, obesity has also been linked to reduced vagal activity,^67–69^ which may explain the observed local hypersensitivity of β-cells to direct cholinergic stimulation. Elevated IL-1β during HFD feeding^70,71^ may enhance vagal tone and β-cell sensitivity to acetylcholine as a compensatory mechanism to mitigate hyperglycemia. Speaking in favor of such a cascade are the observations from β-cell specific cholinergic receptor M3R-overexpressing mice, which have elevated insulin release and are protected from HFD-induced hyperglycemia and glucose intolerance.^3^ These results suggest that increased β-cell sensitivity to cholinergic signaling may initially protect from obesity-driven metabolic dysfunction.

In addition to altering vagal tone, HFD feeding also changes the transcriptional landscape of endocrine cells, wherein diet-induced obesity drives substantially greater transcriptional changes in β-cells than in α-cells.^72^ This aligns with our observation that cholinergic and IL-1β stimulation drastically augments insulin, but not glucagon, secretion from islets isolated from HFD-fed mice. Furthermore, the disruption in intra-islet α-to β-cell communication may contribute to the impaired glucagon-stimulated insulin secretion observed *in vivo*. Tong et al.^49^ demonstrated that despite total GLP-1R expression increasing in β-cells under HFD, the GLP-1R nanodomains enriched at the α-β-cell interface are lost upon metabolic stress. Since these nanodomains are required for GLP-1R pre-internalization and β-cell priming at fasting glucose, their loss could explain why exogenous glucagon failed to stimulate insulin secretion in our HFD model, even as β-cells retained their responsiveness to IL-1β and carbachol stimulation *ex vivo*. The disproportionately elevated response to cholinergic stimulation may also reflect HFD-driven β-cell proliferation, which reaches a peak after short-term HFD-feeding.^73,74^ Finally, we cannot exclude that HFD alters the timing of β-cell responsiveness. Gao et al.^73^ demonstrated that HFD mice develop a progressively delayed first-phase insulin secretion. A similar temporal shift may apply to glucagon-stimulated insulin secretion, the response in our HFD model may have occurred outside the measurement window (1min after i.v. injection) rather than being completely absent.

Our data establish that α-cells play a crucial role in integrating neuronal and immune cues to regulate insulin secretion. This aligns with growing evidence implicating α-cells in β-cell modulation.^22–25,47,49^ Specifically, we propose that α-cells are essential for neuronally driven insulin secretion at fasting glucose levels, possibly to prevent hypoglycemia. Indeed, only in the presence of functional α-cells capable of releasing glucagon can insulin stimulation occur near the threshold of hypoglycemia. More broadly, our study illustrates the importance of immune-neuro-metabolism as an integrated regulatory system.

### Limitations of the study

Compared to humans, mice exhibit more pronounced parasympathetic innervation of the pancreas.^75^ Furthermore, human pancreatic α-cells secrete acetylcholine in order to prime the responsiveness of β-cells, whereas rodent α-cells do not produce acetylcholine.^33^ Thus, the relative contribution of parasympathetic innervation versus islet-derived acetylcholine in humans remains to be determined. In addition, in our *ex vivo* assays, we treated islets with carbachol to model parasympathetic input. While this approach enabled us to model neuronal and IL-1β-stimulated insulin secretion at fasting glucose levels *in vitro*, the carbachol concentration used is supraphysiological (500μM) and does not fully recapitulate the pulsatile nature of *in vivo* cholinergic stimulation. Finally, the contribution of pancreatic δ-cell-derived somatostatin was not assessed in our study. Mouse δ-cells express both muscarinic M3 and M4 receptors, which are coupled to G_q_ and G_i_ G protein-coupled receptors, respectively, exerting opposing effects on somatostatin secretion.^76^ However, carbachol has been shown to inhibit rather than stimulate somatostatin release from isolated mouse islets,^77^ suggesting that suppressed somatostatin tone under our experimental conditions is unlikely to confound with the glucagon and insulin responses we observed in our study.

## RESOURCE AVAILABILITY

### Lead contact

Requests for further information and resources should be directed to and will be fulfilled by the lead contact, Kelly Trimigliozzi (k.trimigliozzi@unibas.ch).

### Materials availability

This study did not generate new unique reagents.

## STAR★METHODS

## EXPERIMENTAL MODEL AND STUDY PARTICIPANT DETAILS

### Animals

All mice were on a C57BL6/N genetic background unless otherwise stated. Female wildtype mice originated from in-house breeding or were purchased from Charles River. α-KO mice^37^ on a C57BL6/N background and wildtype littermates received diphtheria toxin injections to induce α-cell ablation. The *Ins1Cre-GCaMP6^fl/fl^* mice^39^ were hosted at the Center de Recherche du CHUM animal facility Montreal Canada as per Salazar et al.^78^ Manipulations with these mice received approval from the local ethical committee of the CRCHUM (CIPA 2022-10,040 CM21022GRs). All other mice were housed at the specific-pathogen-free (SPF) animal facility at the University Hospital Basel, Switzerland. All animals were housed in a temperature-controlled room with 12h light and 12h dark cycle. All animals had free access to food and water. Mice within an age range of 10–18 weeks were used for experiments unless otherwise stated. All animal experiments in Switzerland were conducted according to the Swiss Veterinary Law and Institutional Guidelines and were approved by the Swiss Authorities. For high-fat diet (D12492, ssniff Spezialitäten; 60% fat, HFD) experiments, mice were fed an HFD starting at the age of 8-week.

### METHOD DETAILS

#### Diphtheria toxin injections

To induce α cell ablation, Diphtheria Toxin (D0564; Sigma) was dissolved in 0.9% NaCl and administered to 5-6-week-old α-KO mice and their wildtype littermates. Three i.p. injections of 500ng each on days 1, 3 and 5 were applied as described by.^48^ The total amount of Diphtheria Toxin each mouse received was 1.5μg. Experiments were conducted 4 weeks after Diphtheria Toxin administration.

#### Cephalic phase experiment

For cephalic phase insulin release, experiments were performed as described.^15^ The mice were fasted overnight (16h) and placed into a new cage with free access to water the following morning. Control mice were placed into an empty cage and kept fasted (control condition), while cephalic-stimulated mice were placed into a cage containing a single food pellet (cephalic condition). Cephalic-treated mice were allowed to approach and take an audible bite of the food pellet and were immediately after restrained and blood glucose was measured, as well as tail blood collected for further analysis. Blood glucose was measured using a glucometer (Freestyle, Abbott Diabetes Care).

#### Glucagon-stimulation test

After an overnight fast (16h), animals were weighed and placed under a redlight heat lamp in order to dilate the tail vein. Mice received an i.v. injection of saline or glucagon (1mg/kg body weight, Sigma) and 1 min after injection, tail vein blood was sampled and blood glucose was measured. For pretreatment with atropine (5mg/kg body weight) or exendin-9-39 (70nmol/kg BW), mice received a prior i.p. injection of saline or atropine/Ex-9-39 30min before an i.v. injection of saline or glucagon. Blood glucose was measured as described above.

#### Acute IL-1β stimulation

Mice were injected intraperitoneally with saline or IL-1β (1μg/kg body weight). After 15min, mice were restrained and tail-vein blood was collected for further analysis. Blood glucose was measured as described above. For pretreatment with atropine (5mg/kg body weight), mice received a prior i.p. injection of saline or atropine 15min before subsequent i.p. injection of saline or IL-1β and a total of 30min prior to blood sampling.

#### Islet isolation

Islets were isolated as described by Steiger et al.^79^ Pancreata were clamped just below the common bile duct and 2-3mL of HBSS collagenase (1.4g/L collagenase type 4; Worthington) was perfused through the sphincter of Oddi. Perfused pancreata were excised and transferred to 50mL tubes containing 2mL HBSS-collagenase solution. Subsequently, the samples were incubated at 37°C for 28min. Samples were homogenized by 15 s of vigorous shaking and washed 3x with HBSS supplemented with 0.5% BSA. Next, samples were filtered through a 500μm mesh and then through an inverted 70μm cell strainer (Corning). Islets were retained on the cell strainer and consequently collected by dipping the cell strainer into a Petri dish containing RPMI-1640 supplemented with 100units/ml penicillin, 100μg/ml streptomycin, 2mM Glutamax, 50μg/ml gentamycin, 10μg/ml Fungison and 10% FCS (Invitrogen). Islets were then handpicked under a light microscope (Nikon Eclipse, TS100) and incubated at 37°C with 5% CO_2_ in supplemented RPMI-1640 solution until further use.

#### *Ex vivo* insulin secretion assays

Following overnight recovery after isolation, mouse islets were pre-incubated for 60–90 min in Krebs Ringer bicarbonate buffer (KRB) supplemented with 115mM NaCl, 4.7mM KCl, 2.6mM CaCl_2_ 2H_2_O, 1.2mM KH_2_PO_4_, 1.2mM MgSO_4_ 7H_2_O, 10mM HEPES, and 0.5% BSA and the pH adjusted to 7.4 and 3.5mM glucose. Calcium-free modified KRB consisted of 115mM NaCl, 4.7mM KCl, 1.2mM KH_2_PO_4_, 1.2mM MgSO_4_ 7H_2_O, 10mM HEPES, and 0.5% BSA with the pH adjusted to 7.4 and 3.5mM glucose. Borosilicate tubes containing 1mL of KRB with 3.5mM or 16.7mM glucose were prepared and 10 islets were added per tube (3–4 replicates per condition). Islets were incubated at 37°C for 60min after which 500μL of supernatant was collected in order to measure released insulin and 500μL of HCl/EtOH was added to each tube for total insulin content extraction. After vortexing and incubating the samples at −20°C for at least 60min, 200μL of sample were collected for insulin content analysis. All extracted samples were stored at −20°C until further analysis. Fold stimulation was calculated by dividing insulin levels in the supernatant by the mean insulin levels of the control (basal) group supernatant. For experiments with 10μM Adomeglivant (GRA) and 100nM exendin-9-39, islets were first pre-incubated with the compounds for 30min.

#### Ca^2+^ imaging of isolated islets

Islets of male *Ins1Cre-GCaMP6^fl/fl^* mice aged 12–18 weeks old were isolated by pancreatic perfusion as described by Ravier and Rutter.^80^ In brief, pancreata were perfused with collagenase-solution (Sigma) into the common bile duct and excised pancreata were incubated for 10min at 37°C in a water bath. After digestion, samples were homogenized by 15 s of vigorous shaking and washed 2x with KRB supplemented with 120mM NaCl, 4.8mM KCl, 2.5mM CaCl_2_, 1.2mM MgCl_2_, 24mM NaHCO_3_, 5mM HEPES, 100 IU Penicillin-Streptomycin, pH adjusted to 7.4, 10mM glucose, 0.5% BSA and 0.01% DNase (Sigma). Next, islets were extracted by density gradient centrifugation. In essence, homogenized pancreata were resuspended in 6mL of 1.119g/L histopaque (Sigma). The following gradient was then layered on top; 1.083g/L histopaque, 1.077g/L histopaque and KRB respectively. Samples were then centrifuged at 800g for 20min with no break. Islets were collected from the upper most layer between KRB and 1.077g/L and washed once with KRB by adding 20mL and centrifuging at 300g for 5min. Supernatant was removed and the pellet containing islets was resuspended in 10mL of RPMI-1640 supplemented with 10% FCS and incubated at 37°C with 5% CO_2_ overnight. Islets were then handpicked and pre-incubated in modified KRB (130mM NaCl, 3.6mM KCl, 0.5mM NaH_2_PO_4_, 1.5mM CaCl_2_, 24mM NaHCO_3_ and 10mM HEPES; pH 7.4) complemented with 3.5mM glucose at 37°C with 5% CO_2_ for 20min. After incubation, islets were moved to glass bottom petri dishes (MaTek) coated with Poly-D-Lysine (Gibco) and modified KRB buffer for imaging. Live-cell imaging of islets was performed using a LSM900-Airyscan (Zeiss) microscope equipped with heated sample holder (37°C). For imaging, the Plan-Apochromat 20x/0.8 objective was used and acquisitions were performed by adjusting focal plane (256× 256 pixels (532.4167 x 532.4167 μm)), no averaging, frame interval of 234ms and using the 488 laser for GCaMP6f excitation for a total run time of 20min. Videos were analyzed using Fiji/ImageJ and mean fluorescence intensity was extracted and processed with *MATLAB*. Normalized fluorescence intensity was calculated by (F-F_min_)/(F_min_), wherein F is the mean fluorescence intensity at the given time point and F_min_ is the lowest intensity recorded during imaging. Time to first response and amplitude of first peak were determined after normalization of Ca2+ traces.

#### Protein measurement assays

Media and plasma insulin was measured using the ultrasensitive mouse/rat insulin kit (#K152BZC-1, MesoScale Discovery). Glucagon in media was measured with the glucagon kit (#KT150U5D, MesoScale Discovery). Plasma glucagon levels were determined using a glucagon ELISA (#10-1271-01, Mercodia).

### QUANTIFICATION AND STATISTICAL ANALYSIS

Statistical analyses were performed using GraphPad Prism (Version 10). No statistical methods were used to predetermine sample size. Data are presented as mean ± standard deviation (SD). The statistical tests used for each experiment, the exact value of n, as well as what n represents are indicated in the figure legends. For comparisons between two groups, a Mann-Whitney test was used. For multiple group comparisons, a one-way ANOVA or two-way ANOVA with Tukey’s multiple comparison test was applied. A *p*-value <0.05 was considered statistically significant.

## Supplementary Material

1

Supplemental information can be found online at https://doi.org/10.1016/j.celrep.2026.117586.

## Figures and Tables

**Figure 1.**
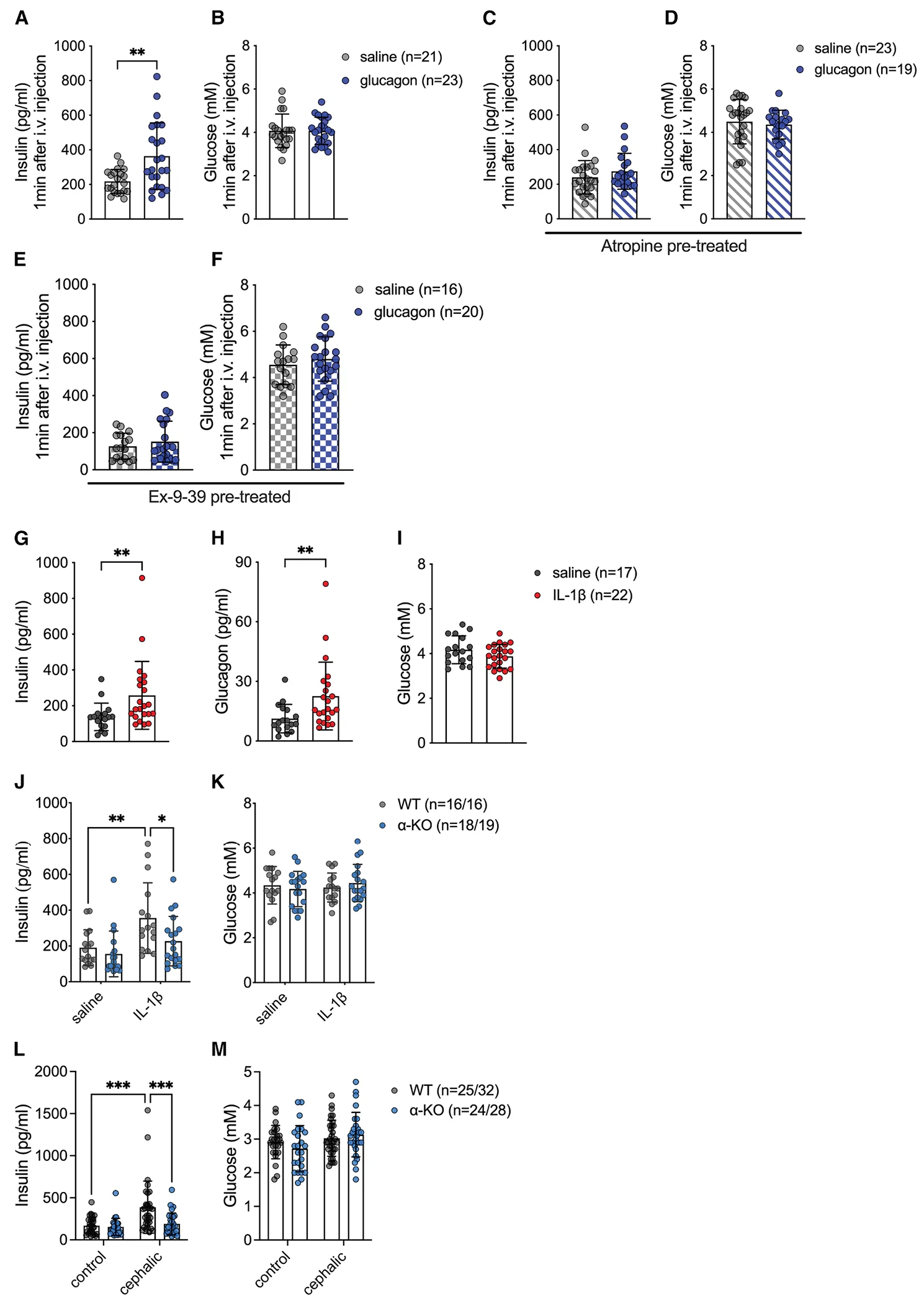
α-cells are essential for neuronally stimulated insulin secretion (A and B) Plasma insulin and (B) blood glucose levels after i.v. injection of saline or glucagon (1mg/kg BW) in overnight-fasted female wildtype C57BL/6N mice. Blood was sampled 1min after i.v. injection. (C and D) Plasma insulin and (D) blood glucose levels in overnight-fasted female wildtype C57BL/6N mice that received an i.p. injection of atropine (5mg/kg BW) 30min before i.v. injection of saline or glucagon (1mg/kg BW). Blood was sampled 1min after i.v. injection. (E and F) Plasma insulin and (F) blood glucose levels in overnight-fasted female wildtype C57BL/6N mice that received an i.p. injection of saline or exendin-9-39 (70nmol/kg BW) 30min before the injection of saline or glucagon (1mg/kg BW). Blood was sampled 1min after i.v. injection. (G–I) Plasma insulin, (H) glucagon, and (I) blood glucose levels of overnight-fasted female wildtype C57BL/6N 15min after i.p. injection of saline or IL-1β (1μg/kg BW). (J and K) Plasma insulin and (K) blood glucose levels of α-KO mice and their wildtype littermates 15min after i.p. injection of saline or IL-1β (1μg/kg BW). For the number of replicates, the format n= (saline/IL-1β) was used. (L and M) Plasma insulin and (M) blood glucose levels after cephalic stimulation of female α-KO mice and their wildtype littermates. For the number of replicates, the format n= (control/cephalic) was used. Data are represented as individual measurements, and bars indicate mean ± SD. n indicates the number of mice. The *p* values of A–I were calculated by the Mann-Whitney Test. For J–M, *p* values were calculated by two-Way ANOVA with Tukey’s multiple comparison test. *p < 0.05, **p < 0.01, ***p < 0.001, and ****p < 0.0001. See also Figure S1.

**Figure 2.**
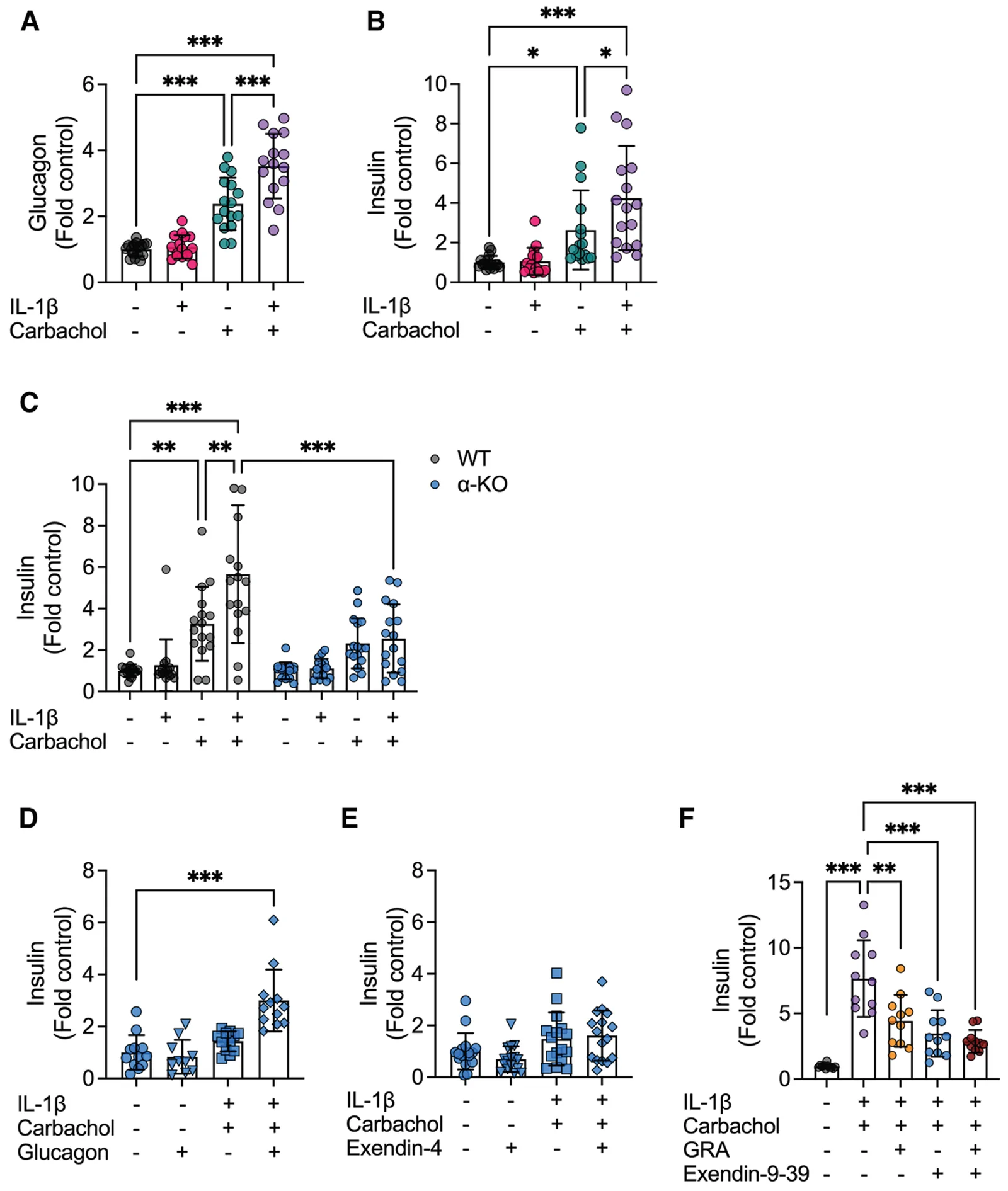
Glucagon facilitates neuronally stimulated insulin secretion *ex vivo* (A and B) Fold stimulation from basal of glucagon and (B) insulin secretion of isolated islets treated with IL-1β (1ng/ml) and carbachol (500μM) at 3.5mM glucose. *n* = 4. (C) Fold stimulation from basal of insulin secretion from isolated islets of α-KO and wildtype littermates treated with IL-1β (1ng/ml) and carbachol (500μM) at 3.5mM glucose. *n* = 4. (D) Fold stimulation from basal of insulin secretion from isolated islets of α-KO mice and their littermate controls treated with IL-1β (1ng/ml), carbachol (500μM), and glucagon (1nM) at 3.5mM glucose. *n* = 4. (E) Fold stimulation from basal of insulin secretion from isolated islets of α-KO mice treated with IL-1β (1ng/ml), carbachol (500μM), and exendin-4 (10nM) at 3.5mM glucose. *n* = 4. (F) Fold stimulation from basal of insulin secretion from isolated islets treated with IL-1β (1ng/ml), carbachol (500μM), Adomeglivant (GRA, 10μM), and exendin-9-39 (100nM) at 3.5mM glucose. *n* = 3 Individual data points represent groups of 10 islets, and bars indicate mean ± SD. n indicates the number of independent experiments. The *p* values of A, B, D, E, and F were calculated by One-Way ANOVA with Tukey’s multiple comparison test. For C, *p* values were calculated by two-Way ANOVA with Tukey’s multiple comparison test. *p < 0.05, **p < 0.01, ***p < 0.001, and ****p < 0.0001. See also Figure S2. Abbreviations: GRA = glucagon receptor antagonist; Ex-9-39 = Exendin-9-39.

**Figure 3.**
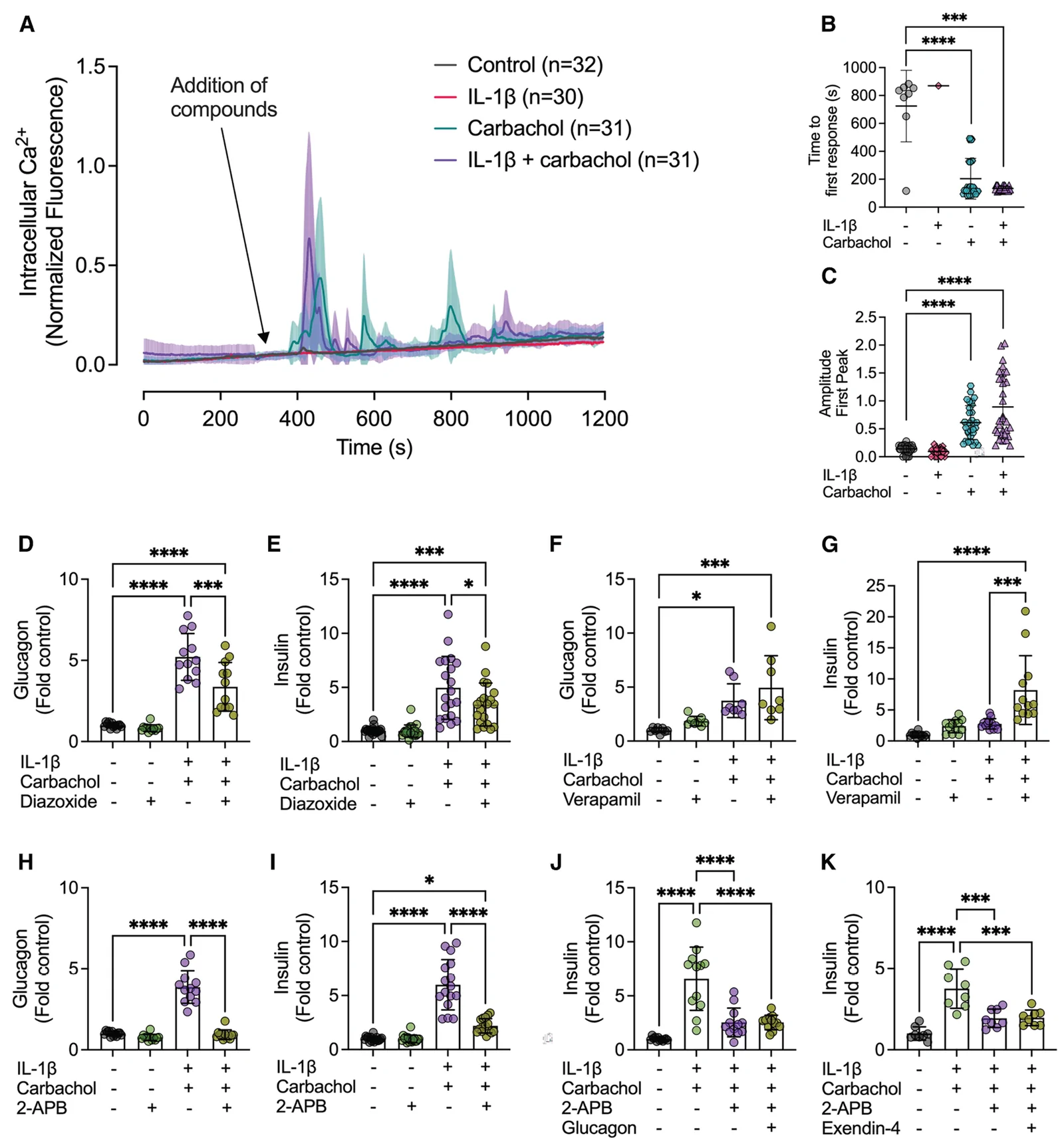
Effects of carbachol and IL-1β on Ca^2+^ dynamics and hormone secretion from isolated islets (A) Average normalized Ca^2+^ traces from islets of *Ins1eGFP-GcAMP6f* mice treated with IL-1β (1ng/ml) and carbachol (500μM). n represents the number of islets recorded. 7–10 islets were recorded per *Ins1eGFP-GcAMP6f* mouse. (B) Time to first response from islets of *Ins1eGFP-GcAMP6f* mice after the addition of IL-1β (1ng/ml) and/or carbachol (500μM). *n* = 30–32 islets per treatment group. (C) Amplitude of first peak from islets of *Ins1eGFP-GcAMP6f* mice after the addition of IL-1β (1ng/ml) and/or carbachol (500μM). *n* = 30–32 islets per treatment group. (D and E) Fold stimulation from basal of glucagon and (E) insulin secretion from isolated islets treated with IL-1β (1ng/ml), carbachol (500μM), and diazoxide (100μM) at 3.5mM glucose. *n* = 5. (F and G) Fold stimulation from basal of glucagon and (G) insulin secretion from isolated islets treated with IL-1β (1ng/ml), carbachol (500μM), and verapamil (100μM) at 3.5mM glucose. *n* = 3. (H and I) Fold stimulation from basal of glucagon and (I) insulin secretion from isolated islets treated with IL-1β (1ng/ml), carbachol (500μM), and 2-APB (100μM) at 3.5mM glucose. *n* = 4. (J) Fold stimulation from basal of insulin secretion from isolated islets treated with IL-1β (1ng/ml), carbachol (500μM), glucagon (1nM), and 2-APB (100μM) at 3.5mM glucose. *n* = 3. (K) Fold stimulation from basal of insulin secretion from isolated islets treated with IL-1β (1ng/ml), carbachol (500μM), 2-APB (100μM), and exendin-4 (10nM) at 3.5mM glucose. *n* = 2. For B-C *p* values calculated by Mann-Whitney Test. For D-K, individual data points represent groups of 10 islets, and n indicates the number of independent experiments. Bars indicate mean ± SD. The *p* values of D–K were calculated by One-Way ANOVA with Tukey’s multiple comparison test. *p < 0.05, **p < 0.01, ***p < 0.001, and ****p < 0.0001. See also Figure S3 and S4. Abbreviations: 2-APB = 2-aminoethoxydiphenyl borate.

**Figure 4.**
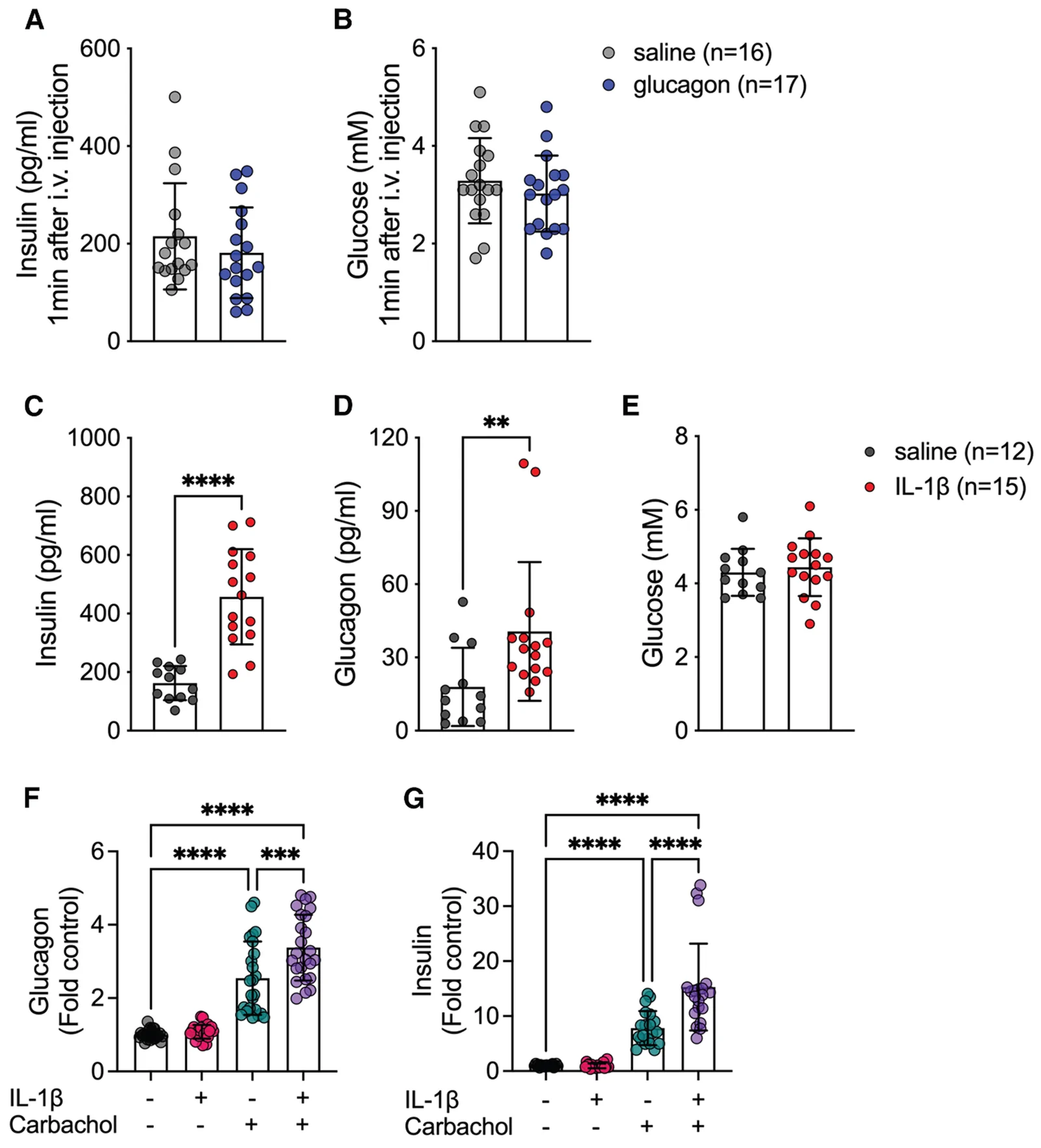
HFD-feeding results in the loss of glucagon-stimulated insulin secretion but sustains IL-1β and carbachol-stimulated insulin secretion *ex vivo* (A) Plasma insulin and (B) blood glucose levels after i.v. injection of saline or glucagon (1mg/kg BW) in overnight-fasted female wildtype C57BL/6N mice after 5 weeks of HFD feeding. (C–E) Plasma insulin, (D) glucagon and (E) blood glucose levels of 5-week HFD-fed female C57BL/6N mice 15min after i.p. injection of saline or IL-1β (1μg/kg BW). (F and G) Fold stimulation of glucagon and (G) insulin secretion of isolated islets treated with IL-1β (1ng/ml) and carbachol (500μM) at 3.5mM glucose. *n* = 6 For A–E data are represented as individual measurements, and bars indicate mean ± SD. For (F and G), individual data points represent groups of 10 islets, and bars indicate mean ± SD. n indicates the number of independent experiments or the number of animals. For A-E, *p* values calculated by the Mann-Whitney Test. For F and G, *p* values were calculated by One-Way-ANOVA with Tukey’s multiple comparison test. *p < 0.05, **p < 0.01, ***p < 0.001, and ****p < 0.0001. See also Figure S5.

**Table:** KEY RESOURCES TABLE

REAGENT or RESOURCE	SOURCE	IDENTIFIER
Chemicals, peptides, and recombinant proteins		
Adomeglivant (GRA)	MCE	Cat#HY-19904
Atropine sulfate salt monohydrate	Sigma	Cat#A0257-5G
Bovine Serum Albumin (Fatty acid free – Low endotoxin)	Sigma	Cat#A8806
Carbachol	Sigma	Cat#PHR1511-500MG
Collagenase Type 4	Worthington	Cat#CLS-4
Collagenase	Sigma	Cat#C9263-500MG
Diazoxide	Sigma	Cat#D9035-1G
Diphtheria Toxin	Sigma	Cat#D0564-1MG
Exendin-4	Sigma	Cat#E7144-.1MG
Exendin-9-39 acetate salt	Bachem	Cat#H-8740.0500
Glucagon	Sigma	Cat#G2044-5MG
Histopaque-1119	Sigma	Cat#11191
Histopaque-1083	Sigma	Cat#10831
Histopaque-1077	Sigma	Cat#10771
Recombinant mouse IL-1β	R&D	Cat#4010-ML
Poly-D-Lysine	Gibco	Cat#A38904-01
Verapamil hydrochloride	Sigma	Cat#V4629-1G
2-APB	EMD Millipore	Cat#100065-100MG
Critical commercial assays		
Mouse/rat insulin kit	Meso Scale Discovery	Cat#K152BZC
Glucagon kit	Meso Scale Discovery	Cat#150U5D
Glucagon ELISA	Mercodia	Cat#10-1271-01
Experimental models: Organisms/strains		
C57Bl6/NCrl *Mus musculus*	In-house breeding (originated from Charles River Laboratories Germany)	MGI:2683688; Strain Code 027; RRID: IMSR_CRL:027
C57Bl6/NCrl mice females	Charles River Laboratories	CRL:027
GluDTR (α-KO)	Thorel et al.^37^	N/A
*Ins1Cre-GCaMP6^fl/fl^*	Delgadillo-Silva et al.^39^	N/A
Software and algorithms		
MATLAB	MathWorks	N/A
Prism Version 10	GraphPad Prism	N/A
Fiji (ImageJ)	N/A	N/A
Other		
High-fat diet	ssniff Spezialitäten, Soest, Germany	Cat# EF D12492 (L) mod. 60kJ %fat [Lard]

## Data Availability

No standardized datasets were generated in this work. This paper does not report original code. Any additional information required to reanalyze the data reported in this paper is available from the lead contact upon request. All data reported in this paper are available from the lead contact upon request.

## Highlights

- α-cells enable immune- and cholinergic-induced insulin secretion at fasting glucose
- Glucagon acts on GCGR and GLP-1R to enable IL-1β and carbachol-driven insulin release
- ER calcium release mediates immune- and cholinergic-stimulated insulin secretion
- High-fat diet renders β-cells sensitive to cholinergic but not glucagon stimulation
