# Supplementary material for: Pancreatic α-cells integrate immune and neuronal stimuli for insulin secretion
Source: Cell Rep. Author manuscript; Available in PMC 2026 Oct 7. (PMC13643029; doi:10.1016/j.celrep.2026.117586)
Supplement: Supplementary file 1 [file NIHMS2199930-supplement-1.pdf]

Cell Reports, Volume *45*

Supplemental information

Pancreatic α-cells integrate immune and neuronal stimuli for insulin secretion

Kelly A. Trimigliozzi, Sophia J. Wiedemann, Giada Ostinelli, Leila Rachid, Hélène Méreau, Marianne Böni-Schnetzler, Guy A. Rutter, Daniel T. Meier, and Marc Y. Donath

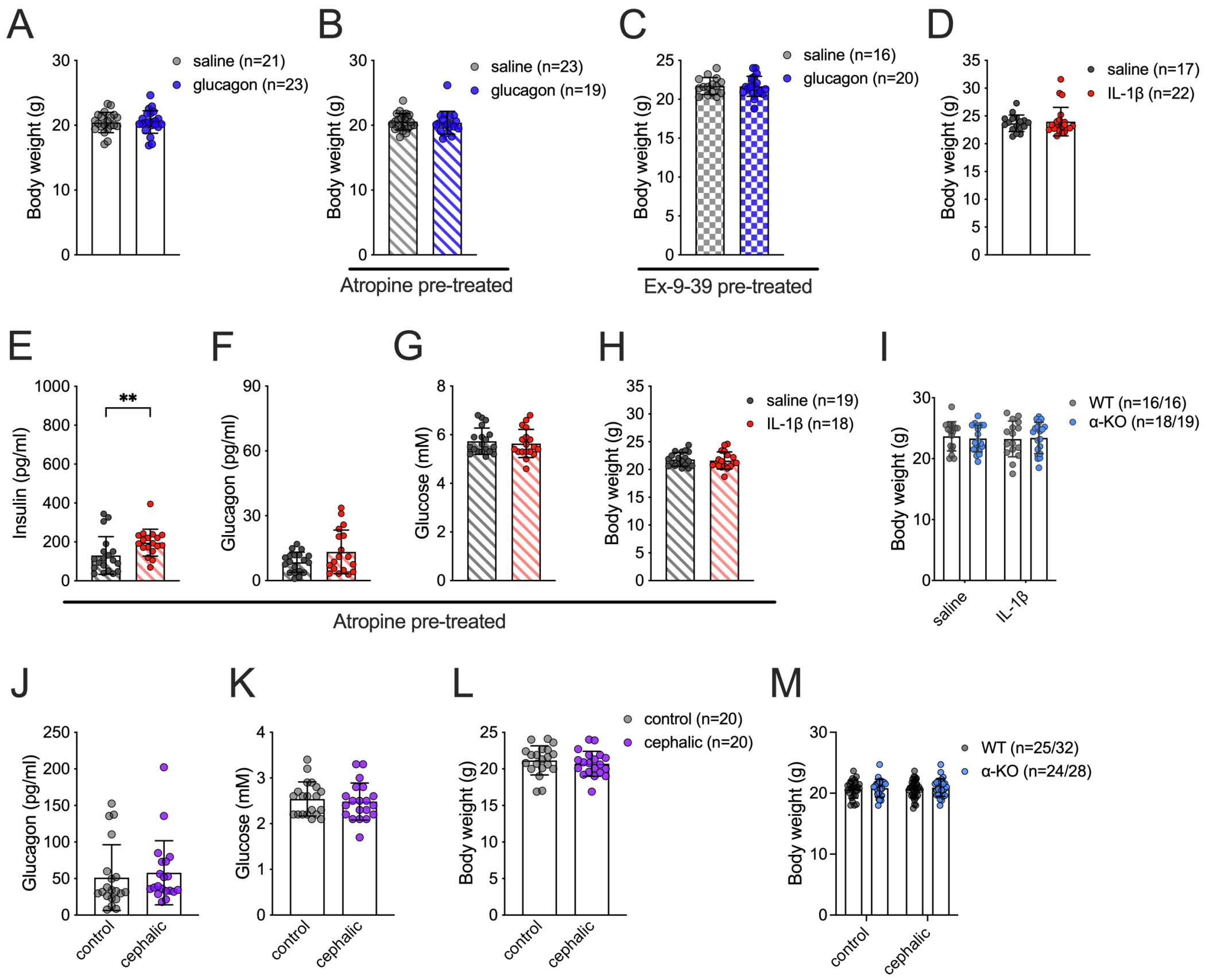

Figure S1. α-cells are essential for neuronally stimulated insulin secretion, Related to Figure 1

(**A**) Body weight of overnight-fasted female wildtype C57BL/6N mice before i.v. injection of saline or glucagon (1mg/kg BW).

(**B**) Body weight of overnight-fasted female wildtype C57BL/6N mice before i.v. injection of saline or glucagon (1mg/kg BW) that were pre-treated with an i.p. injection of saline or atropine (5mg/kg BW) 30min before glucagon/saline injection.

(**C**) Body weight of overnight-fasted female wildtype C57BL/6N mice before i.v. injection of saline or glucagon (1mg/kg BW) that were pre-treated with an i.p. injection of saline or exendin-9-39 (70nmol/kg BW) 30min before glucagon/saline injection.

(**D**) Body weight of overnight-fasted female wildtype C57BL/6N mice before i.p. injection of saline or IL-1β (1µg/kg BW).

(**E**) Plasma insulin, (**F**) glucagon (**G**) blood glucose levels and (**H**) body weight of overnight-fasted female wildtype C57BL/6N mice pretreated with atropine (5mg/kg BW) prior to i.p. injection of saline or IL-1β (1µg/kg BW). Atropine was i.p. injected 30min before blood sampling, and IL-1β/saline was injected 15min before blood sampling.

(**I**) Body weight of α-KO and their wildtype littermates before i.p. injection of saline or IL-1β (1µg/kg BW). For the number of replicates, the format n= (saline/IL-1β) was used.

(**J**) Plasma glucagon, (**K**) blood glucose and (**L**) body weight of female wildtype C57BL/6N mice during cephalic phase stimulation. For the number of replicates, the format n= (control/cephalic) was used.

(**M**) Body weight of α-KO and their wildtype littermates before cephalic stimulation. For the number of replicates, the format n= (control/cephalic) was used.

Data are represented as individual measurements and mean ± SD. The p-values of **A-H, J-L** were calculated by Mann-Whitney Test. For **I** and **M**, p-values were calculated by Two-Way-ANOVA with Tukey's multiple comparison test. *p<0.05, **p<0.01, ***p<0.001, ****p<0.0001

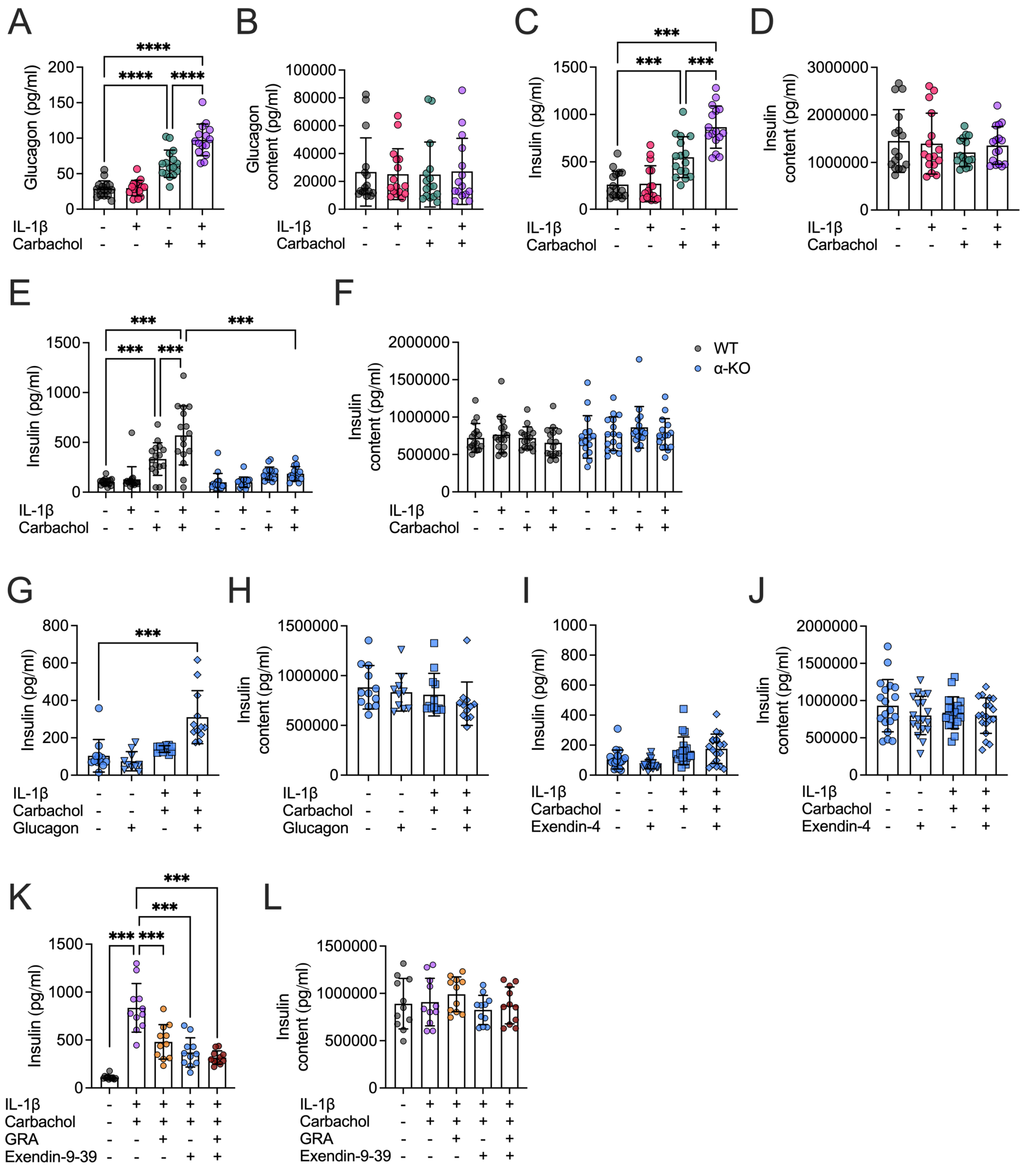

Figure S2. Glucagon facilitates neuronally stimulated insulin secretion *ex vivo*, Related to Figure 2

(**A**) Glucagon in supernatant and (**B**) content of isolated wildtype female islets treated with IL-1β (1ng/ml) and carbachol (500µM) at 3.5mM glucose. n=4.

(**C**) Insulin in supernatant and (**D**) content of isolated wildtype female islets treated with IL-1β (1ng/ml) and carbachol (500µM) at 3.5mM glucose. n=4.

(**E**) Insulin in supernatant and (**F**) content of isolated islets of α-KO mice and their littermate controls treated with IL-1β (1ng/ml) and carbachol (500µM). n=4.

(**G**) Insulin in supernatant and (**H**) content of isolated islets of α-KO mice treated with IL-1β (1ng/ml), carbachol (500µM) and glucagon (1nM) at 3.5mM glucose. n=4.

(**I**) Insulin in supernatant and (**J**) content of isolated islets of α-KO mice treated with IL-1β (1ng/ml), carbachol (500µM) and exendin-4 (10nM) at 3.5mM glucose. n=4.

(**K**) Insulin in supernatant and (**L**) content of isolated wildtype female islets treated with IL-1β (1ng/ml), carbachol (500µM), Adomeglivant (GRA, 10µM) and exendin-9-39 (100nM) at 3.5mM glucose. n=3.

Individual data points represent groups of 10 islets and bars indicate mean ± SD. n indicates number of independent experiments. The p-values of **A-D, G-L** were calculated by One-Way ANOVA with Tukey's multiple comparison test. For **E** and **F**, p-values were calculated by Two-Way-ANOVA with Tukey's multiple comparison test. *p<0.05, **p<0.01, ***p<0.001, ****p<0.0001

Abbreviations: GRA = Glucagon receptor antagonist, Ex-9-39 = Exendin-9-39

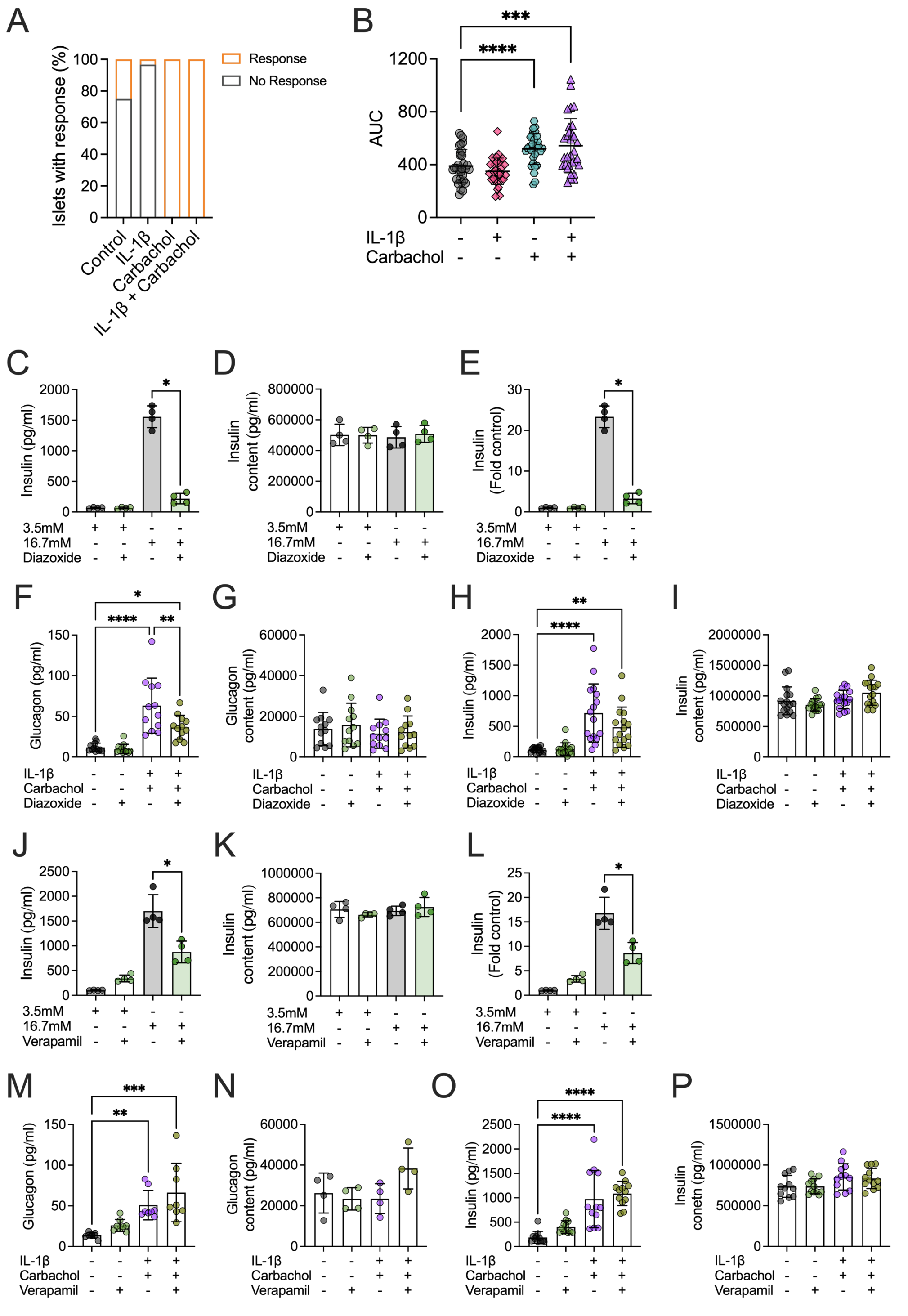

Figure S3. Carbachol and IL-1β-induced insulin secretion does not rely on extracellular calcium influx, Related to Figure 3

(**A**) Percentage of responsive and non-responsive *Ins1eGFP-GcAMP6f* islets treated with IL-1β (1ng/ml) and carbachol (500μM). n=30-32 islets per treatment group.

(**B**) Area under the curve of Ca^{2+} traces of islets treated with IL-1β (1ng/ml) and carbachol (500μM) of *Ins1eGFP-GcAMP6f* mice. n=30-32 islets per treatment group.

(**C**) Supernatant, (**D**) content and (**E**) fold stimulation from basal of insulin from isolated islets treated with Diazoxide (100μM) at 3.5mM and 16.7mM glucose. n=1.

(**F**) Glucagon in supernatant and (**G**) content of isolated islets treated with IL-1β (1ng/ml), carbachol (500μM) and Diazoxide (100μM) at 3.5mM glucose. n=3.

(**H**) Insulin in supernatant and (**I**) content of isolated islets treated with IL-1β (1ng/ml), carbachol (500μM) and Diazoxide (100μM) at 3.5mM glucose. n=5.

(**J**) Supernatant, (**K**) content and (**L**) fold stimulation from basal of insulin from isolated islets treated with Verapamil (100μM) at 3.5mM and 16.7mM glucose. n=1.
(**M**) Glucagon in supernatant and (**N**) content of isolated islets treated with IL-1β (1ng/ml), carbachol (500μM) and Verapamil (100μM) at 3.5mM glucose. n=2.
(**O**) Insulin in supernatant and (**P**) content of isolated islets treated with IL-1β (1ng/ml), carbachol (500μM) and Verapamil (100μM) at 3.5mM glucose. n=3.
For **C-P** individual data points represent groups of 10 islets and bars indicate mean ± SD. n indicates number of independent experiments.
For **B-E**, **J-L** p-values were calculated by Mann-Whitney Test. For **F-I** and **M-P** p-values were calculated by One-Way ANOVA with Tukey's multiple comparison test. *p<0.05, **p<0.01, ***p<0.001, ****p<0.0001

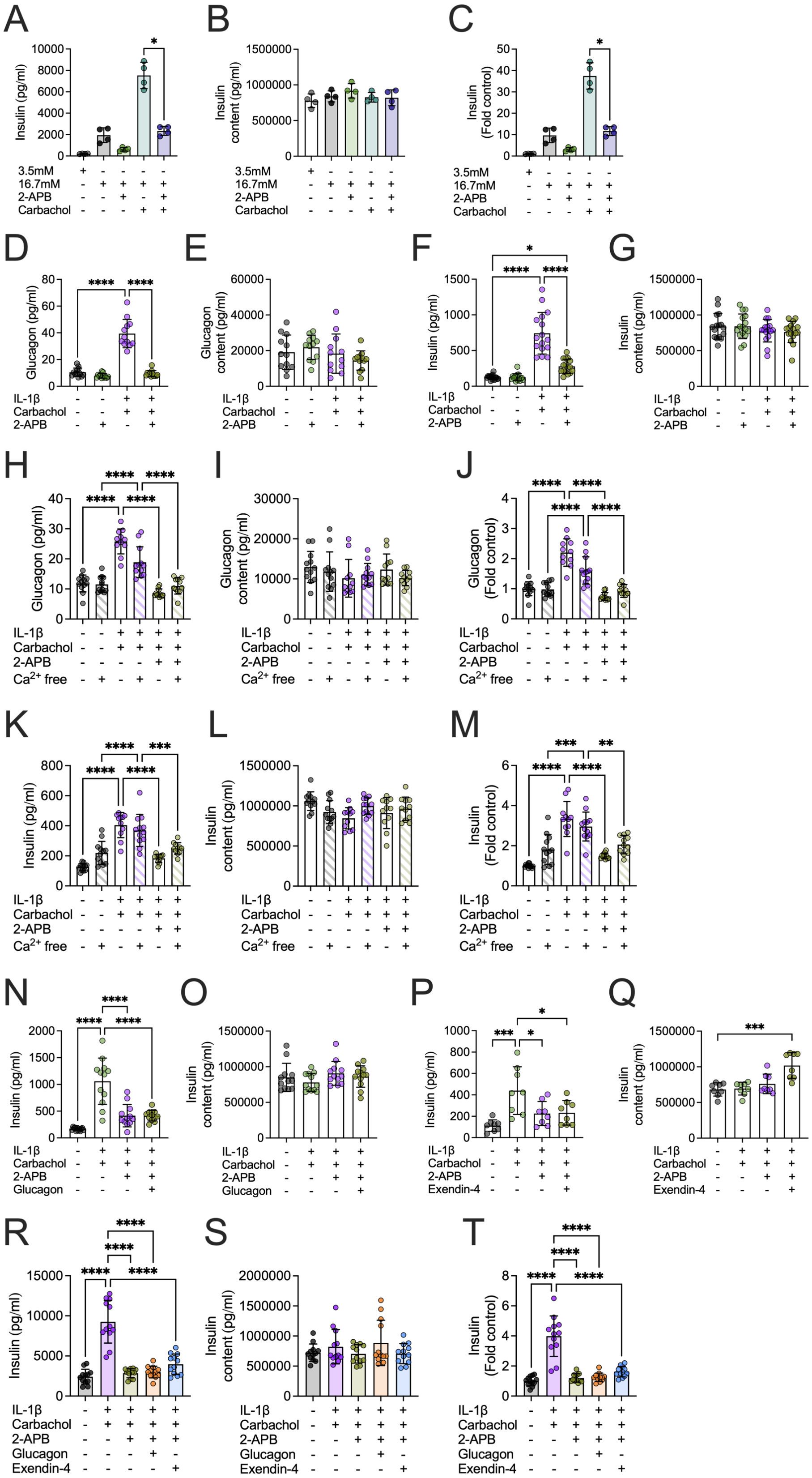

Figure S4. Carbachol- and IL-1β-induced insulin secretion stimulates the release of intracellular calcium stores, Related to Figure 3

(**A**) Supernatant, (**B**) content and (**C**) fold stimulation from basal of insulin from isolated islets treated with 2-APB (100μM) and carbachol (500μM) at 3.5mM and 16.7mM glucose. n=1.

(**D**) Glucagon in supernatant and (**E**) content of isolated islets treated with IL-1β (1ng/ml), carbachol (500µM) and 2-APB (100µM) at 3.5mM glucose. n=3.
(**F**) Insulin in supernatant and (**G**) content of isolated islets treated with IL-1β (1ng/ml), carbachol (500µM) and 2-APB (100µM) at 3.5mM glucose. n=4.
(**H**) Supernatant, (**I**) content and (**J**) fold stimulation from basal of glucagon from isolated islets treated with IL-1β (1ng/ml), carbachol (500µM) and 2-APB (100µM) at 3.5mM glucose in control and Ca^{2+}-free media. n=3.
(**K**) Supernatant, (**L**) content and (**M**) fold stimulation from basal of insulin from isolated islets treated with IL-1β (1ng/ml), carbachol (500µM) and 2-APB (100µM) at 3.5mM glucose in control and Ca^{2+}-free media. n=3.
(**N**) Insulin in supernatant and (**O**) content of isolated islets treated with IL-1β (1ng/ml), carbachol (500µM), 2-APB (100µM) and glucagon (1nM) at 3.5mM glucose. n=3.
(**P**) Insulin in supernatant and (**Q**) content of isolated islets treated with IL-1β (1ng/ml), carbachol (500µM), 2-APB (100µM) and exendin-4 (10nM) at 3.5mM glucose. n=2.
(**R**) Supernatant, (**S**) content and (**T**) fold stimulation from basal of insulin from isolated islets treated with IL-1β (1ng/ml), carbachol (500µM), 2-APB (100µM), glucagon (1nM) and exendin-4 (10nM) at 16.7mM glucose. n=3.
Individual data points represent groups of 10 islets and bars indicate mean ± SD. n indicates number of independent experiments.
For **A-C** p-values were calculated by Mann-Whitney Test. For **D-T** p-values were calculated by One-Way ANOVA with Tukey's multiple comparison test. *p<0.05, **p<0.01, ***p<0.001, ****p<0.0001

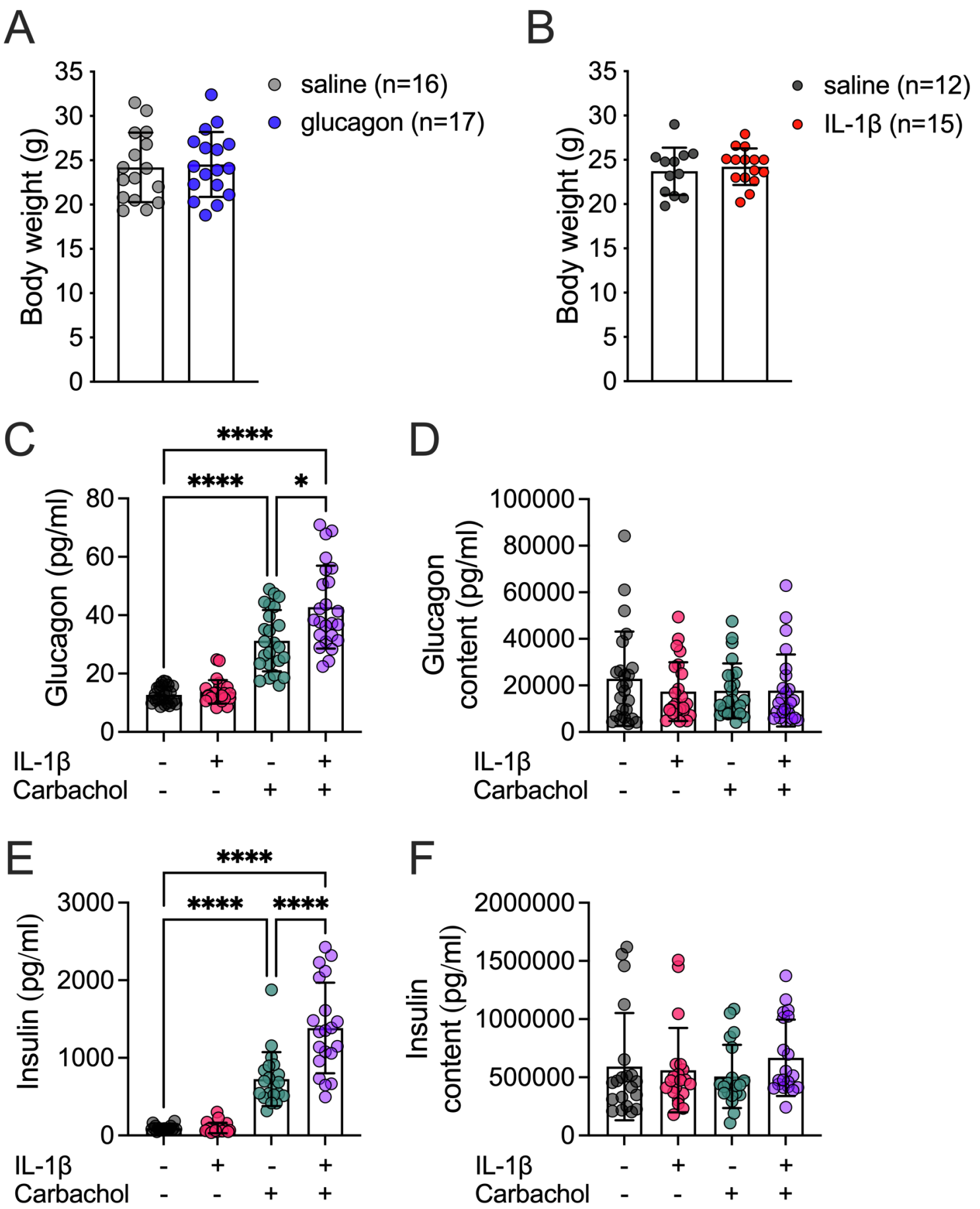

Figure S5. HFD-feeding results in the loss of glucagon-stimulated insulin secretion but sustains IL-1β- and carbachol-stimulated insulin secretion *ex vivo*, Related to Figure 4

(**A**) Body weight of 5-week HFD-fed female wildtype C57BL/6N mice before i.v. injection of saline or glucagon (1mg/kg BW).

(**B**) Body weight of 5-week HFD-fed female wildtype C57BL/6N mice before i.p. injection of saline or IL-1β (1µg/kg BW).

(**C**) Glucagon in supernatant and (**D**) content of isolated wildtype HFD-fed female islets treated with IL-1β (1ng/ml) and carbachol (500µM) at 3.5mM glucose. n=4.

(**E**) Insulin in supernatant and (**F**) content of isolated wildtype HFD-fed female islets treated with IL-1β (1ng/ml) and carbachol (500µM) at 3.5mM glucose. n=4.

Data are represented as individual measurements and mean ± SD. n indicates number of independent experiments.

The p-values of **A** and **B** were calculated by Mann-Whitney Test. For **C-F** p-values were calculated by One-Way ANOVA with Tukey's multiple comparison test. *p<0.05, **p<0.01, ***p<0.001, ****p<0.0001
